# Modafinil Reduces Neuronal Pyroptosis and Cognitive Decline After Sleep Deprivation

**DOI:** 10.3389/fnins.2022.816752

**Published:** 2022-03-03

**Authors:** Xiangyang Xiong, Yan Zuo, Lu Cheng, Zhenyu Yin, Tianpeng Hu, Mengtian Guo, Zhaoli Han, Xintong Ge, Wenzhu Li, Yan Wang, Dong Wang, Conglin Wang, Lan Zhang, Yaodan Zhang, Qiang Liu, Fanglian Chen, Ping Lei

**Affiliations:** ^1^Department of Geriatrics, Tianjin Medical University General Hospital, Tianjin, China; ^2^Department of Neurology, Tianjin Neurological Institute, Tianjin Medical University General Hospital, Tianjin, China; ^3^Tianjin Neurological Institute, Tianjin, China

**Keywords:** modafinil, sleep deprivation, pyroptosis, inflammasome, synaptic plasticity

## Abstract

Sleep deprivation (SD) induces systemic inflammation that promotes neuronal pyroptosis. The purpose of this study was to investigate the effect of an antioxidant modafinil on neuronal pyroptosis and cognitive decline following SD. Using a mouse model of SD, we found that modafinil improved learning and memory, reduced proinflammatory factor (IL-1β, TNF-α, and IL-6) production, and increased the expression of anti-inflammatory factors (IL-10). Modafinil treatment attenuated inflammasome activity and reduced neuronal pyroptosis involving the NLRP3/NLRP1/NLRC4-caspase-1-IL-1β pathway. In addition, modafinil induced an upregulation of brain-derived neurotrophic factor (BDNF) and synaptic activity. These results suggest that modafinil reduces neuronal pyroptosis and cognitive decline following SD. These effects should be further investigated in future studies to benefit patients with sleep disorders.

## Introduction

Sleep is a universal physiological phenomenon in a variety of mammals, including humans. A large number of studies have shown that a lack of sleep can harm health. In mammals, long-term sleep deprivation (SD) can lead to inattention, emotional instability (Yoo et al., 2007), increased sensitivity to pain (Alexandre et al., 2017), induction of metabolic and cardiovascular diseases (Broussard et al., 2012; Huang et al., 2020), and immune dysfunction (Bryant et al., 2004). In extreme cases, it can lead to death. While the pace of life has increased, a scientific understanding of healthy sleep management is currently lacking. In addition, most people rely on sedatives and sleep drugs. It is true that sleep drugs can effectively improve sleep in the early stage, but their effects continuously decline, leading to a need for increased drug dosage and long-term use, which leads to drug addiction. Thus, drugs that can not only reduce the incidence of adverse reactions during treatment but also help patients recover quickly are needed.

Modafinil is an arousal enhancer originally approved for the treatment of paroxysmal narcolepsy (Bastuji and Jouvet, 1988). Recently, modafinil was shown to be effective in treating Parkinson’s disease (Adler et al., 2003), attention-deficit/hyperactivity disorder (Turner et al., 2004), depression, and drug addiction (Martinez-Raga et al., 2008; Kaser et al., 2017). Moreover, modafinil can protect hippocampal neurons by inhibiting excessive autophagy and apoptosis in mice subjected to SD (Cao et al., 2019). Most recently, the effect of modafinil on changes in lipid composition in the brain was studied in Drosophila melanogaster by mass spectrometry imaging. Modafinil was found to decrease the contents of phosphatidylcholine and sphingomyelin and increase the contents of phosphatidylcholine and PI. It was found that modafinil enhances attention and improves learning, memory, and cognitive function (Philipsen et al., 2021). However, to date, the mode of action of modafinil is not completely clear, and whether modafinil exerts effects similar to those of antioxidants to effectively regulate neuronal inflammation in the brain after SD and modulates neuronal pyroptosis after SD is unknown. Therefore, in this study, we hypothesized that modafinil can alleviate neuroinflammation and cognitive impairment such as learning and memory deficits in mice subjected to SD by inhibiting neuronal pyroptosis.

## Materials and Methods

Adult male C57BL/6 mice (10 weeks old, weighing 20–25 g) were purchased from the Chinese Academy of Military Sciences (Beijing). All animal husbandry and experimental procedures complied with the newly revised regulations on the management of experimental animals issued by the State Science and Technology Commission on March 1, 2017, and were performed in accordance with a protocol approved by the Animal Protection and use Committee of Tianjin Medical University.

### Sleep Deprivation Model

**FIGURE 1:**
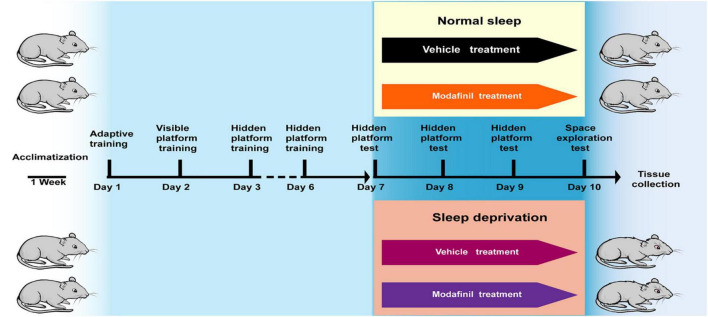
The schematic representation of the experimental design. In the Morris Water Maze (MWM) test, mice were trained for adaptability on the first day, followed by formal training for 2–6 days, positioning navigation testing for 7–10 days (also at the SD stage), and additional space exploration testing after positioning navigation testing on the 10th day. The aim of testing all mice on the morning of day 7 was to exclude those with large individual differences. At the end of the day, they were randomly divided into four groups: the normal group, the Control + MD group, the SD group, and the SD + MD group; subsequently, the sleep deprivation stage was initiated, and the Control + MD group and SD + MD group were treated with modafinil. After the last spatial exploration test, all mice were sacrificed for tissue collection.

The treadmill SD model selected in this experiment was first established at the United States Naval School of Aeronautics (WEBB, 1957). This method has been continuously improved, and scientists have gradually recognized that the experimental results are relatively reliable and that this method decreases the amount of stress imposed on the tested animals (Xu et al., 2010). This method causes much less damage to animals than a traditional water environment or electrical stimulation. Based on the above factors, the treadmill SD method was used to establish an animal model of acute SD (DB036, Beijing). The treadmill conveyor belt was divided into equal-sized squirrel cages with Plexiglas (30 cm × 30 cm × 40 cm). A fence-type squirrel cage lid that was able to feed and water bottles was fastened to the top of the cage, and the treadmill conveyor belt formed the bottom of the squirrel cage. The mice were given free access to food and water while exercising on the treadmill and housed in a quiet animal room on a 12/12 h light/dark cycle with lights on from 6: 00 to 18: 00 at a temperature of 20–22°C and a humidity of 60–65%. The treadmill speed was set to 2.5 m/min, the running time was 3 s, and the rest time was 12 s. The mice were made to run and stop on a cycle. To study the effect of modafinil on the learning and memory of mice subjected to SD, 36 mice were randomly divided into four groups: the control group, SD group, and SD + modafinil (13 mg kg^–1^) group. Studies have shown that modafinil can effectively alleviate learning and memory deficits induced by SD in mice at three doses (6.5, 13, and 26 mg kg^–1^) (Cao et al., 2019). The mice in the control group were not given any treatment, the mice in the SD + modafinil group and Control + modafinil group were given 13 mg kg^–1^ intragastrically, and the mice in the SD group were given the same volume of normal saline for 3 consecutive days as shown in Figure 1.

### Morris Water Maze Test

The Morris Water Maze (MWM) apparatus used in this experiment consisted of four parts: a circular pool (diameter of 120 cm, height of 50 cm), a circular platform (diameter of 12 cm, height of 30 cm), a behavior tracking system and Morris Water Maze analysis software. The experiment was carried out 1 h after intragastric administration as follows: (1) In the positioning navigation test, the circular pool was divided into four equally sized quadrants (I, II, III, and IV) and the duration of the experiment was 90 s. The circular platform was placed in quadrant I, and then the mice were placed in the water close to the pool wall with their backs to the circular platform. The behavior tracking system was started simultaneously. Morris Water Maze analysis software was used to record the swimming time, swimming distance, and swimming trajectory of the mice in searching for the circular platform over 90 s. If a mouse could not find the target platform within 90 s, it was manually guided to the platform and kept there for 15 s. Each animal was trained four times a day with an interval of at least 30 min between the two sessions for 6 consecutive days. The mice were blow-dried immediately after each training and returned to the cage. SD began on the seventh day and lasted for a total of 3 days, and formal tests were conducted on the 10th day. (2) In the spatial exploration test, the experimental parameters were the same as those used in the positioning navigation test except that the circular platform was removed. The experimenter placed the mice in the water close to the pool wall with their backs to the circular platform. At the same time, the behavior tracking system was activated, and Morris Water Maze analysis software was used to record the number of times the mice crossed the location of the circular platform, the amount of time spent in the target quadrant, the swimming speed, the total swimming distance, and the swimming trajectory over at 90 s. After the last spatial exploration test, all mice were sacrificed for tissue collection.

### qRT-PCR

According to the manufacturer’s instructions, total RNA was extracted from mouse hippocampal tissue using TRIzol reagent (Invitrogen, Carlsbad, CA, United States). The concentration of RNA was quantified with a NanoDrop ND-2000 spectrophotometer (Thermo Fisher Science). A FastKing RT Kit was used for reverse transcription, and the SuperReal PREMix Plus kit was used for qRT-PCR. The amplification conditions for real-time PCR instrument were 95°C for 15 min and 40 cycles of 95°C for 10 s, and 60°C for 32 s. The data were analyzed by the 2^–ΔΔCt^ method.

### Western Blotting

Mouse hippocampal tissues were weighed, and protein extraction buffer was added to each sample at a weight–volume ratio of 1:6 (1 ml RIPA cell lysis buffer was supplemented with 10 μl PMSF and 10 μl protein phosphatase inhibitor). Then, the samples were homogenized with grinding instrument (KZ-III-F; Servicebio; China). The cleavage product was centrifuged at 4°C and 13,200 × *g* for 20 min, and the supernatant was collected. Finally, the protein concentration was determined with a BCA protein detection kit (Thermo, United States). The proteins were then separated by SDS-PAGE (8, 10, or 12%) and transferred onto a PVDF membrane for 1.5 h, and the membrane was blocked with blocking buffer (TBST buffer containing 5% skimmed milk powder) for 2 h. The membrane was incubated overnight with NLRP1 (1:1,000; ab98181; Abcam), NLRP3 (1:250; PA5-20838; Thermo Fisher Scientific), NLRC4 (1:1,000; 06-1125; MilliporeSigma), GSDMD (1:1,000; ab209845; Abcam), ASC (1:1,000; sc-22514-R; Santa Cruz Biotechnology, Dallas, TX, United States), cleaved caspase-1 (1:1,000; 67314; CST), IL-1β (1:1,000; 12242; CST), IL-18 (1:1,000; ab71495; Abcam), brain-derived neurotrophic factor (BDNF) (11,000; OSB00018G; Thermo Fisher Scientific), β-actin (1:5,000; 4971; CST), and GAPDH (1:5,000; 2118; CST) primary antibodies at 4°C. After being washed with TBST, the membrane was incubated with the respective secondary antibodies. For densitometry, a ChemiDo XRS + imaging system (Bio-Rad, CA, United States) was used. The average pixel density of each band was measured using Quantity One software (Bio-Rad, CA, United States).

### Immunofluorescence Staining

The mice were anesthetized by intraperitoneal injection of 5% chloral hydrate. After successful anesthesia, the mice were perfused with 4°C PBS until their livers turned white and then decapitated. After overnight immersion in 4% paraformaldehyde, the mice were fixed and then subjected to gradient dehydration in 15 and 30% sucrose solution. After successful dehydration, the olfactory bulbs and brain stems of the mice were excised, and the brain tissues were embedded in OCT compound. Hippocampal sections were fixed, permeabilized, and incubated with mouse anti-caspase-1 (1:200) and rabbit anti-NeuN (1:200) antibodies at 4°C overnight. The next day, the sections were rinsed with PBS and incubated with a mixture of secondary antibodies (FITC-conjugated goat anti-mouse and TRITC-conjugated goat anti-rabbit) for 1 h at room temperature. Finally, the sections were sealed after incubated with DAPI, and the numbers of cells coexpressing caspase-1 and neurons were counted under an immunofluorescence microscope.

### Golgi Staining

A Golgi staining kit (PK401, FD Neuro Technologies, Inc., United States) was used according to the manufacturer’s instructions. (1) At least 24 h before brain extraction, equal volumes of solutions A and B were mixed, and the mixture was kept at room temperature. (2) For brain extraction, the mice in each group were decapitated. The blood on the surface of the brain was quickly washed away with double distilled water, and the tissues were soaked in the solution A and B mixture. (3) After the tissues were incubated for 6 h or on the next day, fresh solution A and B mixture was added, and the tissues were incubated in the dark at room temperature for 2 weeks. (4) The tissues were transferred to solution C and placed in a dark environment at room temperature for at least 72 h (up to 1 week). The solution was replaced once after 24 h. (5) A cryostat was used to cut the tissues into thick 100 μm sections, and then the sections were placed glass slides and dripped with solution C to unfold them. (6) The slices were dried naturally in a dark and dry environment at room temperature. (7) Then the sections were washed twice with double-distilled water for 5 min each. (8) The slices were placed in a 1:1:2 mixture of solution D, solution E, and double-distilled water for 10 min. (9) Then, the slices were washed twice with double-distilled water for 5 min each. (10) The slices were dehydrated successively in 50, 75, and 95% ethanol for 5 min each, and (11) with anhydrous ethanol four times for 5 min each. (12) The sections were cleared in xylene, three times for 5 min each, and (13) sealed with neutral resin, placed in the dark box to dry, photographed, and analyzed. Finally, ImageJ software was used to observe and record the morphology of dendritic spines of hippocampal CA3 pyramidal neurons, record the number and length of dendritic spines, and calculate the density of the dendritic spines.

### Statistical Analysis

The statistical analysis of all measurement data was carried out by using GraphPad Prism 7 statistical software, and the data are expressed as the mean ± SD. Groups were compared by one-way analysis of variance (ANOVA) or two-way ANOVA. The significance level was set as α = 0.05, and significant differences are expressed as *P*-values (*P < 0.05, ^**^P < 0.01, ^***^P < 0.001, and ^****^P < 0.0001).

## Results

### Modafinil Alleviates Cognitive Impairment in Sleep Deprivation Mice

**FIGURE 2:**
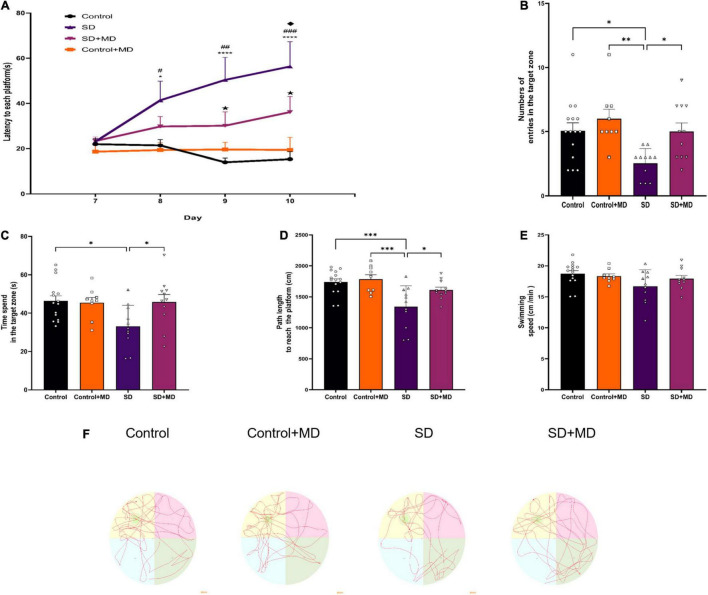
The effect of modafinil on learning and memory of SD. **(A)** The escape latency to reach the platform during the training sessions of the MWM test was detected. *P < 0.05, ^****^P < 0.0001, significantly different from Control; ^#^*P* < 0.05, ^##^*P* < 0.01, ^###^*P* < 0.001, significantly different from Control + MD group; ^*H*^*P* < 0.05, significantly different from SD group; ^*u*^*P* < 0.05, significantly different from SD + MD group. **(B)** The number of entries in the spatial acquisition trial was decreased. **(C)** The time spent in the target quadrant in the probe trail was increased by modafinil treatment. **(D)** The swimming path length was recorded after SD. **(E)** There was no significant difference in swimming speed among the four groups. **(F)** Representative swimming tracks of mice after SD for 72 h. All data presented are means ± SD; *N* = 9–15 mice per group. *P < 0.05, ^**^P < 0.01, ^***^P < 0.001.

Before SD, all mice were subjected to the Morris Water Maze. The mice exhibited the same level of performance in the spatial exploration and positioning navigation tests. As shown in Figure 2A, modafinil treatment alleviated memory deterioration in mice in the hidden platform test (*P* < 0.01). SD obviously impaired the spatial memory of the mice (*P* < 0.001). As shown in Figures 2B–E, after 72 h of SD, the number of times of passing through the hidden platform (*P* = 0.0197), the time of staying in the target quadrant (*P* = 0.0176), and the total distance of swimming (*P* = 0.0005) in the SD group were less than those in the control group. However, after administration of modafinil, the mice in the modafinil group passed through the hidden platform more often (*P* = 0.0401), stayed longer in the target quadrant (*P* = 0.0414), and swam longer (*P* = 0.0447). There was no significant difference in swimming speed among the four groups (*P* > 0.05). The typical swimming trajectories of each group are shown in Figure 2F. Interestingly, although there was a tendency of memory enhancement in the Control + MD group compared with the Control group, there was no statistical significance. Therefore, in this study, we focused on the related mechanisms of modafinil in animals with sleep disorders as opposed to in healthy animals.

### Modafinil Reduces Pyroptosis in Hippocampus Tissues of Sleep Deprivation Mice

**FIGURE 3:**
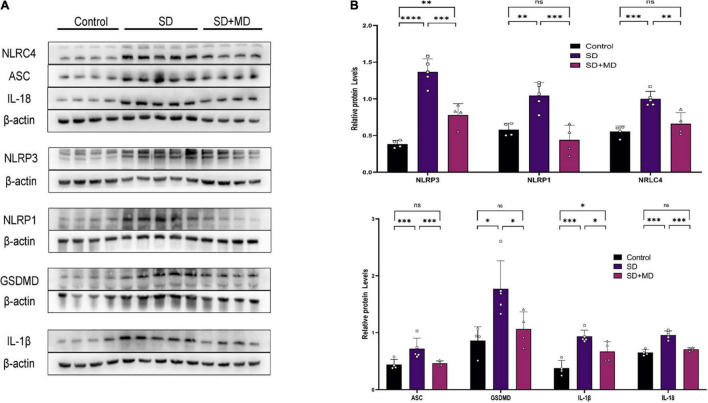
Pyroptosis induced by SD was inhibited by modafinil. **(A,B)** Western blot data showing that modafinil suppressed the increase in the expression of NLRP3, NLRC4, NLRP1, ASC, caspase-1, GSDMD, and the downstream pro-inflammatory cytokines IL-1β and IL-18 in mice subjected to SD. All data presented are means ± SD; *N* = 4–5 mice per group. *P < 0.05, ^**^P < 0.01, ^***^P < 0.001, ^****^P < 0.0001.

As our understanding of pyroptosis has improved, pyroptosis has been gradually associated with the pathophysiological processes of many diseases. However, there are few reports on the relationship between pyroptosis and SD. In this experiment, the expression of many proteins involved in pyroptosis was altered in the hippocampi of SD mice. As shown in Figure 3, the expression of NLRP3 (*P* < 0.0001), NLRC4 (*P* = 0.0004), NLRP1 (*P* = 0.0048), ASC (*P* = 0.0246), GSDMD (*P* = 0.0125), IL-1β (*P* = 0.0004), and IL-18 (*P* < 0.0001) in the hippocampus was increased in the SD group compared with the control group. Modafinil antagonizes the effects of SD on the expression of NLRP3 (*P* = 0.0003), NLRC4 (*P* = 0.003), NLRP1 (*P* = 0.0007), ASC (*P* = 0.0356), GSDMD (*P* = 0.0468), IL-β (*P* = 0.0428), and IL-18 (*P* = 0.0002).

### Modafinil Alleviates Pyroptosis in Hippocampal Neurons of Sleep Deprivation Mice

**FIGURE 4:**
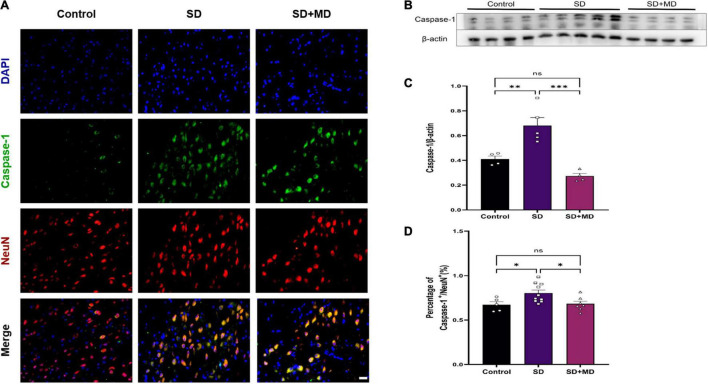
Modafinil decreased the expression of caspase-1 in the hippocampi of mice subjected to SD mice. **(A)** Representative pictures of double staining of caspase-1 and NeuN in the hippocampi of mice obtained by a fluorescence microscope. **(B,C)** Western blot analysis of caspase-1 expression in the hippocampi of mice subjected to SD and treated with vehicle or modafinil. All data presented are means ± SD; *N* = 4–5 mice per group. **(D)** Immunofluorescence analysis and quantification of the expression level of caspase-1. All data presented are means ± SD; *N* = 5–10 mice per group. *P < 0.05, ^**^P < 0.01, ^***^P < 0.001.

We found that the pyroptotic cells in SD model mice were mainly neurons. Next, we evaluated neuronal pyroptosis in mice subjected to SD. Weak caspase-1 immunoreactivity was observed in the normal group, but strong caspase-1 immunoreactivity was observed in the cytosol in mice subjected to SD. The neuronal markers NeuN and caspase-1 were detected by double immunofluorescence. The number of caspase-1-positive neurons in the mouse hippocampus was significantly higher in the group subjected to SD for 72 h than in the control group (*P* = 0.0458). There were significantly fewer caspase-1-positive neurons in the modafinil group than in the SD group (*P* = 0.0392) (Figures 4A,D). This finding was consistent with the western blot results. Caspase-1 expression was significantly increased in the SD group (*P* = 0.0054), and modafinil treatment effectively decreased the expression of this protein (*P* = 0.0003) (Figures 4B,C).

### Modafinil Suppresses Inflammatory Activity in Hippocampus of Sleep Deprivation Mice

**FIGURE 5:**
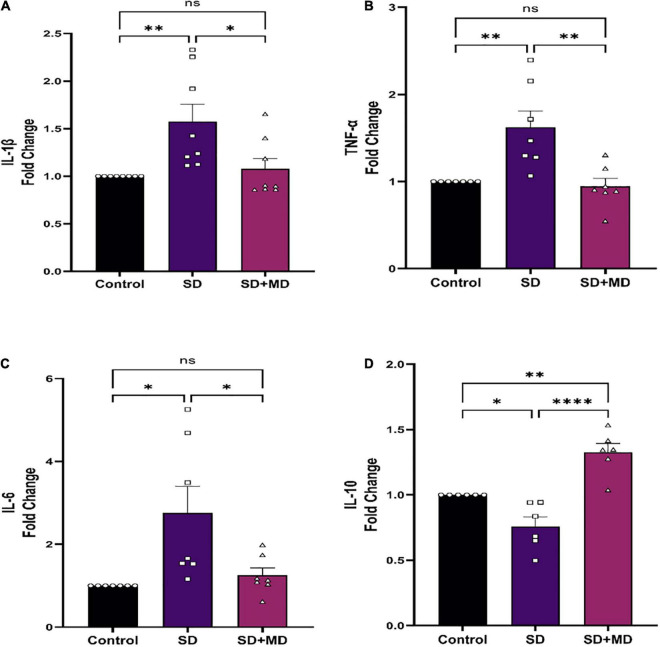
Modafinil altered the expression of inflammatory cytokines in the hippocampi of mice subjected to SD. Modafinil upregulated the expression of IL-1β IL-6 and TNF-α and downregulated the expression of IL-10. All data presented are means ± SEM; *N* = 5–8 mice per group. *P < 0.05, ^**^P < 0.01, ^****^P < 0.0001.

In recent years, the relationship between IL-1β, TNF-α, IL-6, and SD has been widely discussed. It was found that the expression of IL-1β and TNF-α in the serum, heart, liver, kidney, and pancreas is significantly increased in mice subjected to SD (Periasamy et al., 2015). There was a significant correlation between the degree of synaptic damage and synaptic transmission. As shown in Figure 5, qRT-PCR showed that the expression of IL-1β (*P* = 0.0083) (Figure 5A), TNF-α (*P* = 0.0043) (Figure 5B), and IL-6 (*P* = 0.0119) (Figure 5C) was increased and that the expression of IL-10 (*P* = 0.0246) (Figure 5D) was decreased in the hippocampus in mice subjected to SD compared with control mice. Accordingly, modafinil decreased the expression of IL-1β (P = 0.023) (Figure 5A), TNF-α (*P* = 0.0021) (Figure 5B), and IL-6 (*P* = 0.0319) (Figure 5C) and increased the expression of IL-10 (*P* < 0.0001) (Figure 5D) in the mouse hippocampus after SD.

### Modafinil Promotes Brain-Derived Neurotrophic Factor Expression and Synaptic Plasticity in Hippocampus of Sleep Deprivation Mice

**FIGURE 6:**
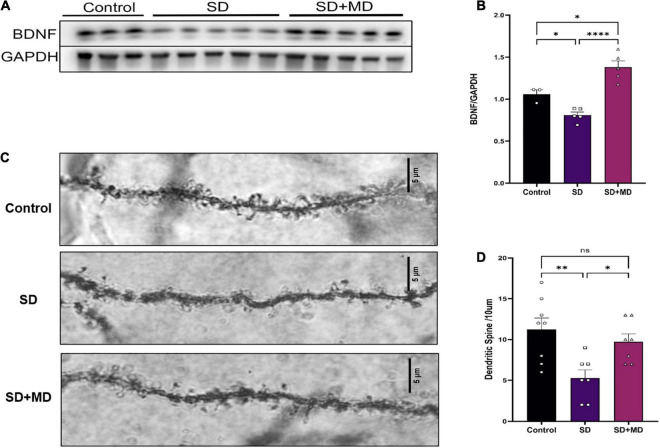
Modafinil decreased BDNF expression and alleviated dendritic spine loss in hippocampal CA3 pyramidal neurons in mice subjected to SD. **(A,B)** Western bot analysis of BDNF expression in the hippocampus. All data presented are means ± SD; *N* = 3–5 mice per group. **(C,D)** The density of CA3 pyramidal neurons, as measured by Golgi staining, was decreased in mice subjected to SD, and modafinil reversed this decrease. All data presented are means ± SD; *N* = 7–8 mice per group. *P < 0.05, ^**^P < 0.01, ^****^P < 0.0001.

Brain-derived neurotrophic factor is thought to be the most important neurotrophin in the central nervous system and is associated with learning and memory (Parkhurst et al., 2013). As shown in Figures 6A,B, Western blotting showed that the protein expression of BDNF in the hippocampus was significantly decreased after 72 h of SD (*P* = 0.0481). The protein expression of BDNF in the hippocampus was significantly increased in the modafinil group compared with the model groups (*P* = 0.0001). Similarly, as shown in Figures 6C,D, dendritic spines on neurons in the CA3 region of the hippocampus were abundant and highly dense in the control group. The density of dendritic spines on neurons in the CA3 region was significantly decreased in the SD group (*P* = 0.0045). Compared with that in the SD group, the density of dendritic spines on hippocampal CA3 neurons in the modafinil group was increased (*P* = 0.0416).

## Conclusion

**FIGURE 7:**
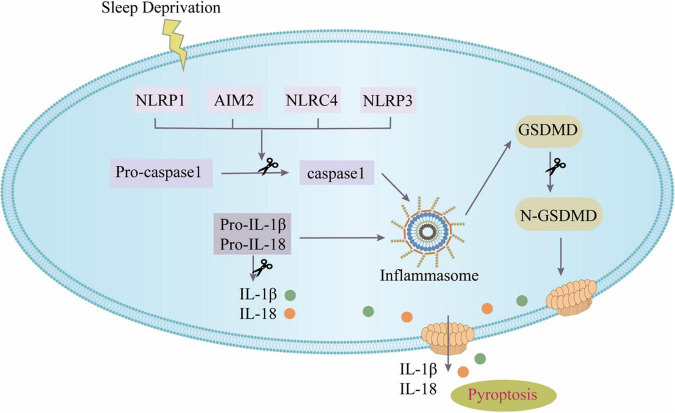
The canonical inflammasome pathway. SD can activate inflammasomes in cells and further recruit ASC and pro-caspase-1 to form inflammasome complex. Active caspase-1 cleaves GSDMD to produce GSDMD pores on the cell membrane; active caspase-1 can activate pro-IL-1β and pro-IL-18, and then IL-1β and IL-18 are released from the GSDMD pores.

This study focused for the first time on the role and mechanism of pyroptosis mediated by the NOD-like receptors (NLRs) inflammasome in SD as shown in Figure 7. The major discoveries are that: (1) modafinil alleviates NLRs inflammasome-mediated pyroptosis in mice subjected to SD; (2) modafinil alleviates inflammation induced by neuronal pyroptosis in mice subjected to SD; (3) modafinil promotes BDNF activation in the hippocampi of mice subjected to SD, which is beneficial for synaptic plasticity; and (4) modafinil improves learning and memory in mice subjected to SD. In summary, targeting the regulation of impaired neuronal pyroptosis and neuroinflammation may be a promising therapeutic strategy for the future treatment of SD.

The term “pyroptosis” was originally to describe a particular type of regulatory cell death (Cookson and Brennan, 2001). That is somewhat similar to apoptosis but is dependent on the inflammatory molecule caspase-1 (Galluzzi et al., 2018). Pyroptosis has been a hot topic in recent years, and an increasing number of studies have shown that it is closely related to a variety of diseases. Pyroptosis is widely involved in intestinal diseases (Bulek et al., 2020), liver diseases (Liu et al., 2020), kidney diseases (Komada and Muruve, 2019), hematological diseases (Johnson et al., 2018), nervous system diseases (Feng et al., 2020), atherosclerotic diseases (Fidler et al., 2021), cancer, and metabolic diseases (Sharma and Kanneganti, 2021). Inflammasome activation is also a key process in severe COVID-19 (Vora et al., 2021). Consistent with previous studies, SD does induce the activation of pyroptosis, but research in this area is relatively insufficient (Fan et al., 2021). Unlike in previous studies, modafinil was able to protect hippocampal neurons by inhibiting excessive autophagy and apoptosis in sleep-deprived mice (Cao et al., 2019). In this study, modafinil inhibited the further activation of pyroptosis and reduced cognitive impairment in sleep-deprived mice. In-depth study of pyroptosis is helpful for elucidating its role in the occurrence, development and prognosis of related diseases and provides new ideas for clinical prevention and treatment.

Early research has shown that modafinil has a variety of positive effects on awakening, movement, and cognitive ability (Minzenberg and Carter, 2008). The new study also found that modafinil enhances attention and improves learning, memory, and cognitive function (Philipsen et al., 2021). At the same time, SD inhibits the expression of BDNF in the hippocampus, which in turn disrupts synaptic plasticity, leading to neurologic decline in the hippocampus and, ultimately, a decline in learning and memory (Zagaar et al., 2016). However, the mechanism has not been fully identified. In this study, an inflammatory response that inhibited the expression of BDNF and the development of synaptic plasticity in the hippocampus was found to be activated in sleep-deprived mice. Modafinil reduced further inflammation, boosting BDNF activation in the hippocampus and synaptic plasticity in mice. Moreover, behavioral tests showed that modafinil significantly alleviated learning and memory impairment in sleep-deprived mice, possibly through inhibition of neuronal pyroptosis and inflammatory activation.

Studies have shown that skeletal muscle, as an endocrine organ, can release many muscle cytokines during exercise and play an anti-inflammatory role (Hoffmann and Weigert, 2017). As a simple and convenient aerobic exercise, treadmill exercise can inhibit neuroinflammation and microglial activation (Mee-Inta et al., 2019). Treadmill exercise can also prevent inflammation and learning and memory impairment caused by acute SD and reverse the cognitive decline caused by SD (Kojima et al., 2020). In addition, treadmill exercise reduced chronic allergic lung inflammation and airway remodeling in mice (Vieira et al., 2007). Treadmill exercise could increase myeloid-derived suppressor cells (MDSCs) by stimulating the secretion of IL-10 from macrophages through the IL-10/STAT3/S100A9 signaling pathway, thereby achieving heart protection (Feng et al., 2021). Depression symptoms were alleviated by reducing the number of microglia and inhibiting microglial activation and neuroinflammation in the hippocampus. Treadmill exercise lessens hepatic inflammation during non-alcoholic steatohepatitis by reducing the accumulation of hepatic monocyte-derived inflammatory macrophages and bone marrow precursor cells (Fredrickson et al., 2021). Treadmill exercise plays a beneficial role in promoting neurogenesis and functional recovery by activating the CD200/CD200R signaling pathway and improving the inflammatory environment after stroke (Sun et al., 2019). These studies suggest that treadmill exercise has a favorable effect on the balance between pro- and anti-inflammatory and reinforce its potential therapeutic role in reducing the risk of neuroinflammation-related diseases. However, it takes a long time for treadmill exercise to exert its anti-inflammatory effects. In this experiment, intermittent and brief treadmill exercise was mainly used to disturb the sleep of mice, and whether it affected the inflammatory process needs further study.

In conclusion, our study demonstrates that modafinil suppresses neuronal pyroptosis and inflammation following SD. The potential benefit of modafinil in patients with sleep disorders may deserve further investigation in future studies.

## Data Availability Statement

The datasets presented in this study can be found in online repositories. The names of the repository/repositories and accession number(s) can be found in the article/supplementary material.

## Ethics Statement

The animal study was reviewed and approved by the Animal Protection and Use Committee of Tianjin Medical University. Written informed consent was obtained from the owners for the participation of their animals in this study.

## Author Contributions

PL and YZu were responsible for study design. XX developed methodology. XX, YZu, LC, ZY, TH, MG, ZH, XG, WL, YW, and DW carried out the experiments. FC and QL provided technical support. XX and YZu interpreted the results, performed data analysis, and prepared the figures and tables. XX wrote the manuscript. PL supervised the study. All authors read and approved the final manuscript.

## Conflict of Interest

The authors declare that the research was conducted in the absence of any commercial or financial relationships that could be construed as a potential conflict of interest.

## Publisher’s Note

All claims expressed in this article are solely those of the authors and do not necessarily represent those of their affiliated organizations, or those of the publisher, the editors and the reviewers. Any product that may be evaluated in this article, or claim that may be made by its manufacturer, is not guaranteed or endorsed by the publisher.
